# Supplementary material for: Multifunctional Biomimetic Composite Coating with Antireflection, Self-Cleaning and Mechanical Stability
Source: Nanomaterials (Basel). 2023 Jun 13;13(12):1855. doi: 10.3390/nano13121855 (PMC10300925; doi:10.3390/nano13121855)
Supplement: Supplementary file 1 [file nanomaterials-13-01855-s001.zip › nanomaterials-2428774-supplementary.pdf]

nanomaterials

MDPI

Supporting Information

Supplementary Materials S1: The Influence of SiO_2/PDMS/Matte Polyurethane Composition Ratio on Coating Properties

S1.1. Effect of SiO_2 NPs content on reflectivity and wettability of composite coatings

For the coatings synthesized by adding 2.5 g matte polyurethane and 0.5 g curing agent, SiO_2 NPs of different masses and 0 g PDMS, the influence of different masses of SiO_2 NPs on the reflectance and wettability of the coating surface was discussed. It can be seen from Figure S1a that the aluminum alloy after surface treatment has a high reflectivity, about 55%-75%. When the surface of the aluminum alloy is only sprayed with a matte polyurethane coating (without adding SiO_2 NPs), the surface reflectance is reduced to about 20.91%. However, when a certain amount of SiO_2 NPs was added to the coating, the surface reflectivity was further reduced. It shows that both the matte polyurethane and the SiO_2 NPs contribute to the antireflective property of the coating surface. Specifically, with the increase of the amount of SiO_2 added, the surface reflectance of the coating showed a trend of increasing gradually. When the amount of SiO_2 added is 0.5 g, the minimum reflectance of the coating surface is about 12.17%, and when the amount of SiO_2 increases to 2.0 g, the minimum surface reflectance reaches about 16.21%. In particular, when SiO_2 is 1.5 g, the lowest reflectivity is about 13.47%, which is very close to the reflectance of the coating surface when SiO_2 is added at 0.5 g.

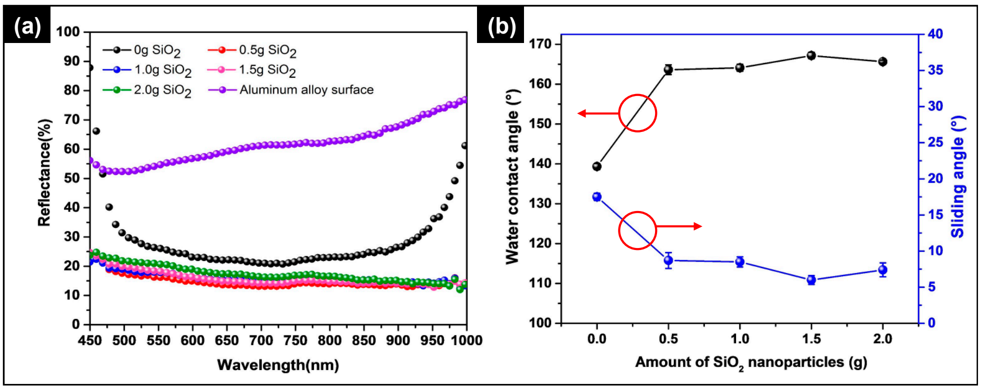

Figure S1. The effect of different SiO_2 NPs additions on the surface reflectance and wettability of the coating. The curves of (**a**) reflectance and (**b**) CA and SA of the coating surface with different additions of SiO_2.

In addition, it can be seen from Figure S1b that the coating without SiO_2 displays a hydrophobic state. Its CA is 139.33 ± 0.75°, and the SA is 17.5 ± 0.5°. However, when SiO_2 NPs were introduced, the wettability of the coating surface changed from hydrophobic to superhydrophobic state. In other words, with the gradual increase of SiO_2, the CA shows a trend of increasing first and then decreasing. In particular, when the amount of SiO_2 added is 1.5 g, the CA of the coating surface reaches the maximum, which is 167.18 ± 0.80°. In addition, the doping of SiO_2 with different qualities makes the SA decreases first and then increase. When SiO_2 is 1.5 g, the SA is the smallest, which is 6 ± 0.61°. The above results show that matte polyurethane and modified SiO_2 NPs can effectively improve the antireflection properties and surface wettability of aluminum alloy surfaces at the same time. SiO_2 NPs play a crucial role in realizing these two properties of the composite coating. Therefore, when the addition amount of SiO_2 is 1.5 g, the coating surface can obtain the best reflectivity and wettability.

S1.2. Effect of PDMS content on reflectivity and wettability of composite coatings

For the composite coatings synthesized by adding 2.5 g matte polyurethane, 0.5 g curing agent, 1.5 g SiO_2 NPs and different mass of PDMS, the effects of PDMS content ratio on the reflectance and wettability of the coating were mainly discussed. As shown in Figure S2a, compared with the

coating without PDMS, the antireflective property of the coating surface can be effectively improved after the introduction of PDMS. Specifically, with the increase of the amount of PDMS, the reflectivity of the coating surface gradually decreased. When the PDMS is 1 g, the minimum reflectivity of the coating surface is about 19.21%, and when the PDMS is increased to 4 g, the minimum reflectivity reaches about 9.22%.

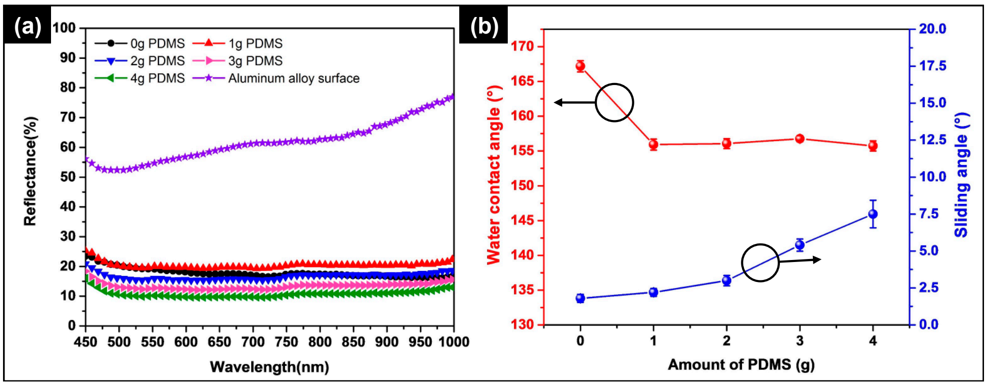

Figure S2. The effect of different PDMS additions on the reflectance and wettability of the coating surface. The curves of (**a**) reflectance and (**b**) CA and SA of the coating surface with different additions of PDMS.

The main reason for this phenomenon is that the introduction of PDMS further increases the agglomeration between structures. Compared with the coating without PDMS, a more compact micro-/nano- composite structure is formed on the surface, facilitating the effect of the surface on incident light. However, the introduction of PDMS has a significant effect on the CA and SA of the coating surface (Figure S2b). Compared with the coating without PDMS (CA of 167.18 ± 0.80° and SA of 6 ± 0.61°), when 1 g of PDMS was added, the surface CA decreased to 155.92 ± 0.80°. With the addition of PDMS, the CA showed a trend of increasing first and then decreasing. When the PDMS is 3 g, the surface CA is the largest, which is 156.77 ± 0.43°. The corresponding SA gradually increases with the increase of PDMS. When 3 g of PDMS was added, the SA of the coating surface reached 5.4 ± 0.42°. The main reason for this phenomenon is that the increasing PDMS is equivalent to adding an "even coating" to the surface of the part, increasing the contact area between the drops and the coating surface and reducing the CA. The above results show that the introduction of PDMS can further improve the antireflective property of the coating surface, and reduce its wettability. Taking the above results into consideration, it can be confirmed that when the addition amount of PDMS is 3 g, the coating surface can show good antireflective and wetting properties at the same time.

S1.3. Effect of matte polyurethane content on reflectivity and wettability of composite coatings

For the BCCs synthesized by adding different mass of matte polyurethane (its mass ratio to curing agent is 5:1), 1.5 g SiO_2 NPs and 3 g PDMS, the effects of adding different mass of matte polyurethane to the BCC were discussed. Influence rule of coating surface reflectivity and wettability. It can be seen from Figure S3a that with the increase of the amount of matte polyurethane added, the reflectance of the coating surface shows a trend of first decreasing and then increasing. When the added amount of matte polyurethane is 4.5 g, the minimum surface reflectance is about 7.64%; when it increases to 5.5 g, the minimum surface reflectance reaches about 11.43%. This is mainly due to the low reflectivity property of the matte polyurethane material, which, combined with the micro/nano hierarchical structure present in the coating, further promotes the effect of the coating surface on incident light. However, with the gradual increase of matte polyurethane, it may cause changes in the surface structure of the coating, resulting in relatively more scattered light on the surface, which increases the reflectivity of the coating surface.

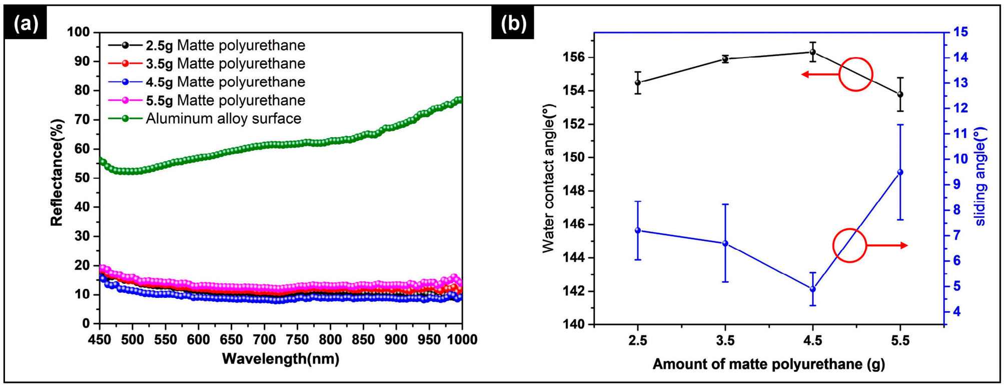

Figure S3. The curve of the influence of different amounts of matte polyurethane on the reflectance and wettability of the coating surface. The curve of the coating surface (**a**) reflectance (**b**) CA and SA with different additions of matte polyurethane.

As for the wettability of the coating, it can be seen from Figure S3b that with the increase of the amount of matte polyurethane added, the surface CA first increases and then decreases. In particular, when the matte polyurethane is 4.5 g, the CA is the largest, which is 156.32 ± 0.58°. At the same time, the SA of the coating surface showed a trend of first decreasing and then increasing, and the minimum SA was 4.9 ± 0.65°. Although the matte polyurethane has hydrophilic properties, the whole system has a superhydrophobic state due to the combined action of PDMS and FAS-17 hydrophobic agent in the hybrid system. However, with the increase of matt urethane, the hydroxyl groups in the hybrid system gradually increased, reducing the wetting performance of the coating. It can be seen that under the premise of other conditions remaining unchanged, the amount of matte polyurethane can affect the surface reflectivity and wetting performance of the entire coating system. At the same time, with the gradual increase of its addition amount, the abrasion resistance of the coating is also improved. Based on the above results, it can be concluded that when the addition amount of matte polyurethane and curing agent is 4.5 g and 0.9 g, the coating surface can achieve excellent antireflection and wetting properties.

Supplementary Materials S2: Morphologies of Biomimetic Coatings with Different Component Ratio

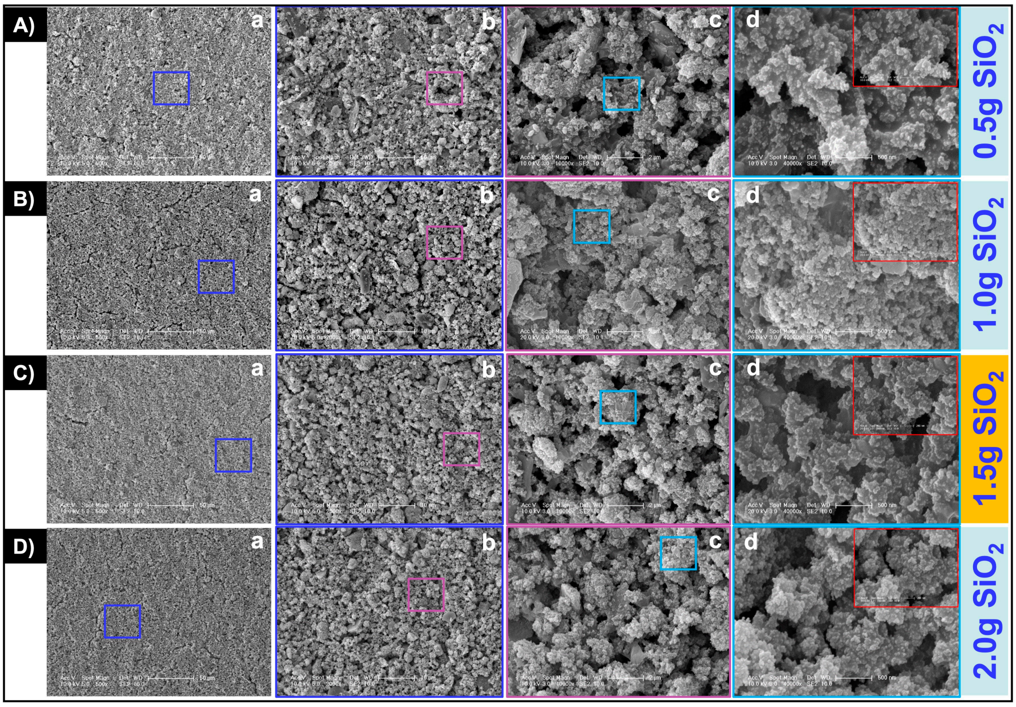

Figure S4. Surface morphologies of coatings with different amounts of SiO_2 NPs added. (**A**) 0.5 g, (**B**) 1.0 g, (**C**) 1.5 g, (**D**) 2.0 g. (**A**–**D**) correspond to the SEM images of different magnifications in turn.

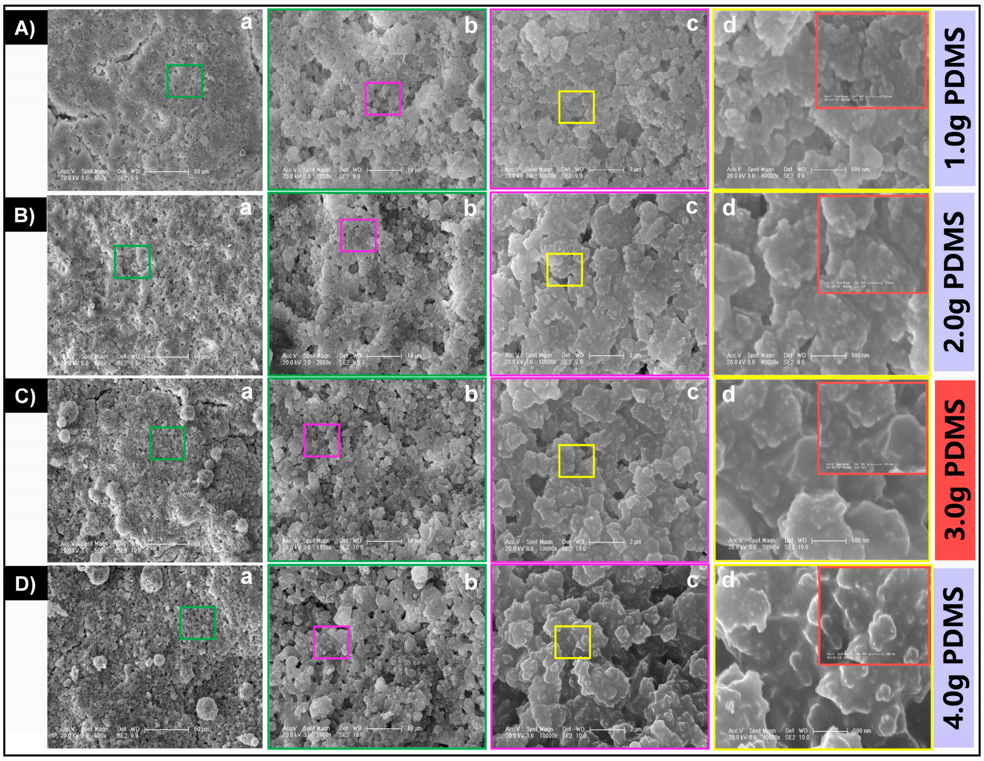

Figure S5. Surface morphologies of coatings with different amounts of PDMS added. (**A**) 1.0 g, (**B**) 2.0 g, (**C**) 3.0 g, (**D**) 4.0 g. Among them, (**A**–**D**) correspond to the SEM images of different magnifications in turn.

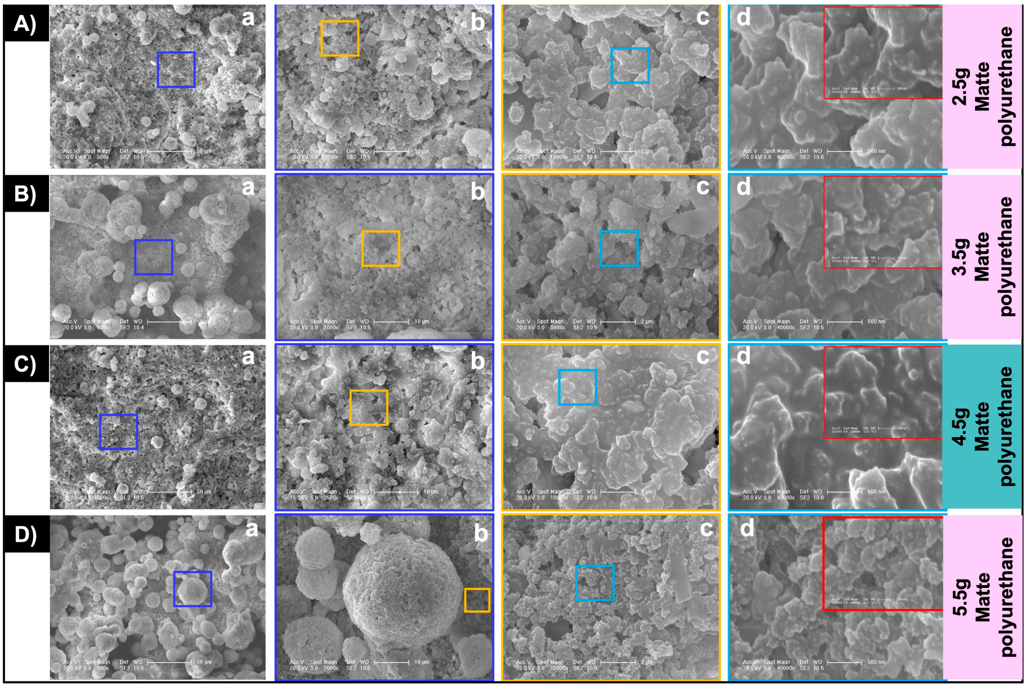

Figure S6. Surface topography of coatings with different amounts of matte polyurethane added. (**A**) 2.5 g, (**B**) 3.5 g, (**C**) 4.5 g, (**D**) 5.5 g, and (**A**–**D**) correspond to the SEM images of different magnifications in turn.

Supplementary Materials S3: Preparation of the SiO_2 NPs

The ethyl orthosilicate, hexamethyldisilazane (HMDS), ammonia, anhydrous ethanol, and deionised water were purchased from Shanghai Aladdin Biochemical Technology Co., Ltd. The alkali-catalyzed preparation of SiO_2 NPs (with a particle size of approximately 200 nm) was carried out as follows:

(1) A mixture of 5.39 g of TEOS and 14.34 g of anhydrous ethanol was added to a round bottom flask and stirred magnetically at 25 °C for 10 min.

(2) Adding a mixture of 14.34 g of anhydrous ethanol, 2 ml of ammonia and 0.93 g of deionized water drop by drop to the above mixture of (1) and react at the developed hydrolysis temperature for 5 h.

(3) Upon completion of the reaction, 8.35 g of HMDS modifier was added to the mixed solution in (2) and stirred magnetically for 3 h.

(4) The resulting solution is aged for 12 h and separated by high-speed centrifugation (10,000 rpm) for 15 min (at least five times), the resulting SiO_2 powder is then filtered and washed with anhydrous ethanol (at least three times) until the solution is neutral (pH = 7).

(5) Subsequently, it is transferred to an oven and dried at 80 °C for 6 h to obtain SiO_2 powder.

(6) Remove the dried SiO_2 powder sample and place it in a grinding bowl to grind it into a fine powder, then, place the powder in the drying oven again and dry it at a temperature of 100°C for 2 h to obtain the modified SiO_2 powder. The modified SiO_2 NPs were given hydrophobic properties due to the replacement of a large number of surface hydroxyl groups by the methyl group of HMDS.
